# Using Patient-Reported Outcomes to Predict Revision Arthroplasty Following Femoral Neck Fracture: Enhancing the Value of Clinical Registries through Data Linkage

**DOI:** 10.3390/ijerph16081325

**Published:** 2019-04-12

**Authors:** Christina L Ekegren, Richard de Steiger, Elton R Edwards, Richard S Page, Raphael Hau, Susan Liew, Andrew Oppy, Belinda J Gabbe

**Affiliations:** 1 Department of Epidemiology and Preventive Medicine, Monash University, Melbourne, VIC 3004, Australia; ere@bigpond.net.au (E.R.E.); raphaelhau@hotmail.com (R.H.); belinda.gabbe@monash.edu (B.J.G.); 2 Epworth Hospital, Richmond, VIC 3121, Australia; richard.desteiger@epworth.org.au (R.d.S.); andrewoppy@me.com (A.O.); 3 Department of Surgery, University of Melbourne, Melbourne, VIC 3010, Australia; 4 Department of Orthopaedic Surgery, Alfred Hospital, Melbourne, VIC 3004, Australia; s.liew@alfred.org.au; 5 Department of Orthopaedics, University Hospital Geelong, Geelong, VIC 3220, Australia; richard.page@deakin.edu.au; 6 Barwon Centre for Orthopaedic Research and Education, School of Medicine, Deakin University, Geelong, VIC 3220, Australia; 7 Department of Orthopaedic Surgery, Box Hill Hospital, Melbourne, VIC 3128, Australia; 8 Department of Orthopaedics, Northern Hospital, Epping, VIC 3076, Australia; 9 Department of Surgery, Monash University, Clayton, VIC 3800, Australia; 10 Department of Orthopaedic Surgery, Royal Melbourne Hospital, Melbourne, VIC 3050, Australia; 11 Health Data Research UK, Swansea University Medical School, Swansea SA2 8PP, UK

**Keywords:** registries, patient reported outcome measures, femoral neck fractures, arthroplasty, pain

## Abstract

The aim of this study was to determine the association between patient-reported outcome measures (PROMs) six months following femoral neck fracture after a low fall and future arthroplasty, and the factors associated with this. Six-month post-fracture PROMs were collected from the Victorian Orthopaedic Trauma Outcomes Registry (VOTOR) for patients aged >55 years who were admitted for a femoral neck fracture after a low fall between March 2007 and June 2015. These cases were linked with those registered by Australian Orthopaedic Association National Joint Replacement Registry (AOANJRR) up to October 2016. Multivariable analysis was performed using a Cox proportional hazards model to determine factors associated with future arthroplasty, including six-month PROMs. Of the 7077 hip fracture patients registered by VOTOR during the study period, 2325 met the inclusion criteria. Internal fixation being used for the initial hip fracture surgery, being younger and having no pre-injury disability were all independently associated with future revision or conversion to arthroplasty. Out of all PROMs, reporting pain and discomfort six months post-fracture was associated with a 9.5-fold increase in the risk of future arthroplasty (95% CI: 3.81, 23.67). The value of clinical registries can be enhanced via data linkage, in this case by using PROMs to predict arthroplasty following femoral neck fracture.

## 1. Introduction

Hip fractures are significant injuries that lead to reduced quality of life and increased mortality, especially in older patients [1,2]. In many countries, ageing populations are giving rise to an increase in hip fracture cases, placing additional burdens on healthcare systems [3]. Improved life expectancies are also expected to increase the likelihood of patients requiring further hip surgery when previous surgical fixation has failed, or when the initial arthroplasty requires revision [4].

Previous research has established that re-operation rates are lower for arthroplasty compared to internal fixation (approximately 10% compared to 40%) [5,6]. However, for younger hip fracture patients, internal fixation is often preferred over arthroplasty as it is associated with less operative trauma, reduced blood loss, and a lower risk of infection. Thus, in order to assess patient risk and guide surgical decision-making on the need for further surgery, it is important to understand factors associated with re-operation beyond just the technique and type of initial hip fracture surgery.

In the past, research on predictive factors for re-operation has relied on demographics, pre-fracture health status and post-operative radiological findings [7,8]. While these factors have relevance, a key limitation is that a patient’s pre-fracture health may be significantly different to their post-fracture health. Furthermore, adverse radiological findings are often well tolerated by patients, while many patients experience severe functional limitations or pain in the absence of any adverse radiological findings [9,10]. As such, understanding patients’ post-fracture symptoms and their association with re-operation can guide patients and clinicians on the need and timing for further surgery.

To assess outcomes that matter most to patients, patient-reported outcome measures (PROMs) are being increasingly adopted following orthopaedic surgery [11]. The inclusion of PROMs within clinical registries offers a time and cost-effective way of following up large cohorts of specific patient groups. However, there is a lack of consensus on which PROMs are most important to include within orthopaedic registries, and when to collect them, in order to optimise patient care [11,12]. By linking a large orthopaedic trauma registry that collects PROMs with a population-level joint replacement registry, this research provided the first opportunity to determine the association between PROMs six months post-injury and future arthroplasty, as well the factors associated with this.

## 2. Materials and Methods

### 2.1. Patients and Setting

All hip fracture patients registered by the Victorian Orthopaedic Trauma Outcomes Registry (VOTOR) from 1 March 2007 to 30 June 2015 were included. The VOTOR is a sentinel site registry which, since March 2007, has included four trauma hospitals within the state of Victoria (population 6.3 million) [13]: two major (level 1) trauma centres, one metropolitan trauma service, and one regional trauma service [14]. All patients aged ≥16 years with an orthopaedic admission >24 h to a VOTOR hospital, excluding those with pathologic fractures, are captured by VOTOR.

To determine the incidence of arthroplasty following hip fracture, the VOTOR hip fracture dataset was linked with the Australian Orthopaedic Association National Joint Replacement Registry (AOANJRR) dataset for the period 1 March 2007 to 31 October 2016. The AOANJRR includes data on almost all arthroplasty procedures performed in Australia since 2002. Data are validated against patient-level data provided by each of the state and territory health departments in Australia with use of a sequential, multilevel matching process [15].

Ethical approval was obtained for this study from each of the four VOTOR hospitals via the multi-site ethics review process (HREC16Alfred/92), as well as the Monash University Human Research Ethics Committee (CF16/2058 - 2016001024). Both VOTOR and the AOAJNRR use an opt-out method of consent, with the opt-out rate currently at <3% for VOTOR, while the AOANJRR has had only 46 patients opt out in 16 years (1.2 million procedures) [14,15].

The study population consisted of all patients registered by VOTOR with a hip fracture who: (i) were aged 55 years and over, (ii) had sustained a fractured neck of femur (as defined by International Classification of Diseases 10th version Australian Modification (ICD-10-AM) diagnostic codes: S72.00–S72.05, S72.08, S72.10) [16], and (iii) were injured via a low fall. We excluded patients injured via high-energy mechanisms, such as road traffic injuries and high falls, due to their different characteristics and outcomes compared to low-fall patients [17].

### 2.2. Procedures

For the included cohort, the following variables were extracted from VOTOR: age at time of hip fracture; gender; date of birth; date of hip fracture; comorbidities (via the Charlson Comorbidity Index (CCI), mapped from ICD-10-AM codes) [18]; date of surgery; surgical procedures (Australian Classification of Health Interventions (ACHI) codes) [19]; pre-injury level of disability (self-reported as none, mild, moderate, marked or severe disability and analysed as “no disability” or “disability present”); and dates of death (obtained via routine linkage between VOTOR and the Victorian Registry of Births, Deaths and Marriages).

Six-month PROMs were extracted from VOTOR for all patients surviving to six months post-injury. These data were captured over the telephone by trained interviewers who contacted patients or their next of kin (where contact with the patient was not possible due to factors such as language other than English, cognitive deficit, etc.). For this study, the following six-month PROMs were included based on their relevance to the hip fracture population: (i) place of residence (“home without care”, “home with care” or “nursing home/other”) and (ii) Three-Level European Quality of Life-Five Dimensions (EQ-5D-3L) measure. The EQ-5D-3L asks patients to score their mobility, activity, pain and discomfort, anxiety and depression, and self-care, as “no problems”, “some problems” or “severe problems” [20]. Adhering to convention, EQ-5D-3L responses were dichotomised into “no problems” and “some problems” [20]. As the EQ-5D-3L was only introduced to the registry in 2009, missing EQ-5D-3L outcome data from 2007 and 2008 were mapped from the Short-Form 12 (SF-12) responses using previously validated methods [21].

### 2.3. Data Linkage

Following VOTOR case selection, unique patient identifiers including name, date of birth, medical record (UR) number and date of index surgery were securely transferred to the AOANJRR, where probabilistic linkage was carried out. Probabilistic linkage takes into account a wide range of potential identifiers (e.g., date of birth, UR number) to ensure a potential case match. When there is a probability above a certain threshold that two given records refer to the same case, the two records are considered to be matches. For each linked case with a future arthroplasty, the following AOANJRR variables were added to the dataset: date, type and diagnosis/reason for arthroplasty, components inserted, bearing surface for components inserted, diameter of femoral head and whether cement was used on the femoral and acetabular side.

After the linked dataset was returned to VOTOR, a medical record review was undertaken for each linked patient at each of the four VOTOR hospitals to determine the laterality of the original hip fracture (i.e., left or right side). This step was necessitated by the reliance of VOTOR on diagnostic coding via the ICD-10-AM, which does not include laterality. Subsequently, fractures were matched with any subsequent arthroplasties according to laterality. The ICD-10-AM does not record fracture displacement, nor did we conduct radiological reviews; therefore, we were unable to determine fracture displacement.

### 2.4. Data Analysis

Descriptive statistics were used to summarise the characteristics of the sample. Chi square analyses were carried out to compare patient characteristics (age, gender, CCI and pre-injury disability) and index procedures between those with complete and incomplete follow-up data. Chi square analyses were also carried out to determine univariable associations between patient characteristics, index procedures, six-month EQ-5D-3L and residential outcomes, and future arthroplasty. A multivariable analysis was performed using a Cox proportional hazards model, where the primary outcome was the time from the initial hip fracture surgery to the first arthroplasty procedure (if the index procedure was internal fixation) or time to the revision arthroplasty (if the index procedure was an arthroplasty) within the follow-up period. Patients were followed until censoring (ranging from 1.3 to 9.7 years after index surgery, corresponding to the date of registry linkage) or until death. The proportional hazards assumption was evaluated statistically based on Schoenfeld residuals and a log–log Kaplan–Meier survival plot against time. The variables included in the multivariable analysis were the significant factors identified in the univariable analysis (*p* < 0.25) [22]. Non-significant variables were removed from the model individually in a backward stepwise approach (*p* < 0.05). The reduced model was compared with the initial model using likelihood ratio tests and the remaining variable coefficients were assessed to ensure that they had not substantially changed, indicating potential confounding. This process was repeated until a parsimonious final model was achieved [22].

To account for the mapping of EQ-5D-3L data from SF-12 data in 2007 and 2008, a sensitivity analysis was carried out comparing results from the unmapped dataset (2009 onwards) to the dataset containing mapped data. All analyses were conducted within Stata (Version 13, StataCorp, College Station, USA) and alpha was set at 0.05.

## 3. Results

**Figure 1:**
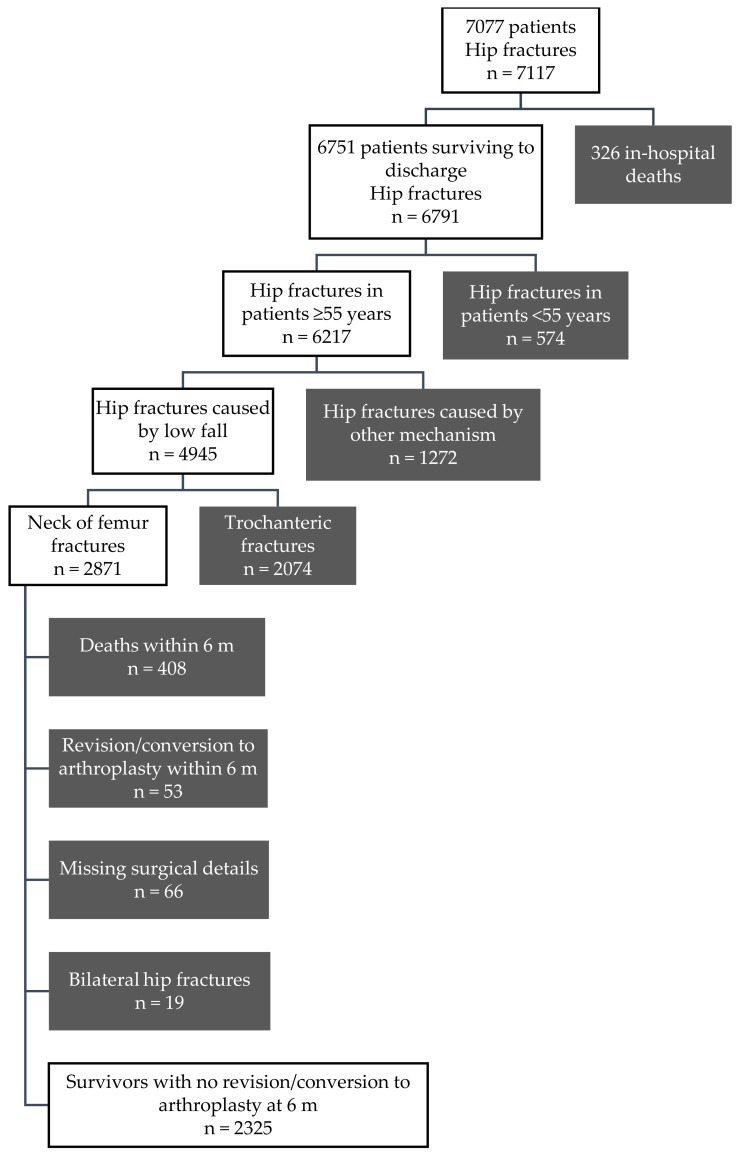
Participant and hip fracture flow chart; m: months after index surgery.

The flow of patients and hip fractures through the study is shown in Figure 1. Out of 7117 hip fractures, 40% met the inclusion criteria (n = 2871). Of these, we excluded 53 patients who required a revision arthroplasty or conversion to arthroplasty within six months of their initial surgery, 408 patients who died within six months, and 66 patients who had missing surgical details. For the 19 patients with bilateral hip fractures we included only their first hip fracture. The characteristics of the remaining 2325 patients are shown in Table 1. Almost three-quarters of included patients were female and 40% were aged 85 years and older. The rate of death subsequent to the first six months was 46% (n = 1079).

Seventy-seven patients required an arthroplasty six months or more after their initial surgery (Table 1). Main reasons for revision or conversion to arthroplasty included failed internal fixation (n = 31), acetabular erosion (n = 13), osteonecrosis (n = 11), prosthesis loosening (n = 9) and infection (n = 3). Patients with the highest rate of revision or conversion to arthroplasty were those who had their initial fracture treated with internal fixation, while those with the lowest rate had undergone cemented hemiarthroplasty (Table 1). Revision or conversion arthroplasties were more common in younger patients who had less pre-injury disability and a lower comorbidity index.

Table 2 presents PROMs for all survivors who had not yet undergone revision arthroplasty or conversion to arthroplasty six months after initial surgery. Six-month PROM response rates ranged from 80% to 91%, depending on the measure. There was no association between incomplete follow-up data at six months and any patient characteristic except for the type of index surgery (Table S1). Living at home, having no problems with personal care, and reporting pain and discomfort six months post-injury were factors associated with a higher rate of future arthroplasty.

Table 3 presents results of the multivariable Cox regression analysis for revision arthroplasty or conversion to arthroplasty. After adjusting for age, pre-injury disability, comorbidity index, six-month residential status, and personal care and pain/discomfort items of the EQ-5D-3L, internal fixation remained a significant predictor of future arthroplasty relative to cemented hemiarthroplasty, as did being younger and having no pre-injury disability. Reporting pain and discomfort at six months was associated with a 9.5-fold increase in the risk of future arthroplasty.

A sensitivity analysis was carried out, comparing these results to those from a subset of data restricted to those patients with a date of injury from 2009 onwards (including only unmapped EQ-5D-3L data) (Table S2). All findings were consistent for this subset of data.

## 4. Discussion

The aim of this study was to link two clinical registries to determine the association between PROMs six months post femoral neck fracture and future arthroplasty, and other factors associated with this. Consistent with previous research, the risk of revision arthroplasty or conversion to arthroplasty six months after initial hip fracture surgery was highest among patients undergoing internal fixation, relative to total hip arthroplasty and hemiarthroplasty. The risk was also higher for patients who were younger and reported no pre-injury disability. Patients reporting pain and discomfort six months post-injury had an almost ten-fold higher risk of future arthroplasty compared to those with no pain.

While there is a small body of literature investigating PROMs as predictors of subsequent revision following arthroplasty, to our knowledge, this is the first study to investigate PROMs predicting arthroplasty following hip fracture. Our major finding was the strong association between pain and discomfort six months post hip fracture and future arthroplasty. Similar to this, previous studies have shown associations between low Oxford Hip Scores (OHSs) (i.e., increased pain and reduced function) at both six months and five years post arthroplasty, and subsequent revision [23,24]. Likewise, patients with moderate to severe pain following total hip arthroplasty were more likely to undergo revision than those with joint stiffness or deteriorating X-rays [25]. In a more recent study, patients taking higher amounts of opioids three to six months post total hip arthroplasty had a higher risk of revision surgery both one and five years later [26]. Importantly, our study found no association between any other six-month outcomes such as mobility, self-care, usual activities or anxiety/depression, and subsequent arthroplasty. Consequently, our findings reinforce the importance of pain as a critical outcome for inclusion in follow-up assessments, and the outcome that matters most to patients in relation to further surgery. Moreover, although recovery from hip fracture can be slow in older adults, this study supports surgical decision-making to revise based on patient symptoms at six months, rather than waiting too long to revise and potentially prolonging patient suffering.

Another key finding was that further surgery was more common in younger patients and those reporting no pre-injury disability. These findings are supported by previous research reporting greater revision rates following arthroplasty in younger patients [27,28,29,30], and suggests that surgeons may be more likely to operate on those with greater functional demands and less surgical risk. Younger patients with less disability may also be more active, leading to excessive strain on the implant or prosthesis [27,31]. Older patients may be more tolerant of a poorly functioning or painful hip as they may have lower functional demands or other more imperative health issues. Furthermore, older patients with cognitive impairments may have less access to healthcare or be unable to voice their concerns, particularly in relation to pain [31].

Notably, after adjusting for confounding, we found no association between pre-injury comorbidity and future arthroplasty. This finding contradicts previous research demonstrating a greater risk of revision in patients with higher degree of pre-injury comorbidity, possibly due to susceptibility to post-surgical infection or poor bone quality [27,32]. The lack of association between comorbidity and future arthroplasty in our study may be explained by our use of the Charlson Comorbidity Index, which reflects a limited number of life-threatening conditions, none of which relate to bone quality [18]. However, while low bone mineral density is a well-defined risk factor for hip fracture, evidence is contradictory regarding its association with the need for revision arthroplasty [8,33,34]. Further research is required to determine the importance of this risk factor for revision following hip fracture.

A key limitation of this study was the small number of patients undergoing internal fixation, as their index procedure may have been revised using further internal fixation surgery or implant removal, and these data were not available. However, revision via arthroplasty is the recommended management for patients over 60 years of age [35], who constituted 97% of our sample, so the number of patients undergoing any other revision procedure was likely to be negligible. Also, because the study was observational, only association was shown and—considering the potential for unexplained confounding—causality cannot be confirmed. Furthermore, owing to our reliance on ICD-10-AM coding within the trauma registry, we did not have any information on fracture displacement, which is an important risk factor for surgical failure following internal fixation [8,29].

Despite these limitations, there were several strengths to our study. Using data from two high-quality registries provided access to PROMs with high follow-up rates in a large volume of hip fracture cases. This provided the first strong evidence of post-fracture PROMs predicting future arthroplasty. These methods also provide access to baseline (pre-revision) PROMs in order to quantify the level of improvement post-revision. Another strength was our use of a standardised and widely-used outcome measure, the EQ-5D-3L. This outcome measure has been recommended for use following both fracture and arthroplasty, and is currently used in several arthroplasty registries [10,12,20]. Our novel finding of pain and discomfort predicting future arthroplasty beyond all other patient-reported outcomes reinforces the importance of the routine collection of PROMs—particularly pain levels—within clinical registries. Furthermore, our successful linkage of two large datasets demonstrates the utility of this method for enhancing the value of clinical quality registries.

## 5. Conclusions

While there has been extensive research investigating predictive factors for revision arthroplasty following femoral neck fracture, these have focused on patients’ pre-fracture health, surgical techniques and radiological findings. This study linked two large clinical registries: an orthopaedic registry collecting PROMs and a national clinical quality registry of joint replacement procedures, providing the first opportunity to determine the association between PROMs six months post femoral neck fracture and future arthroplasty, and the factors associated with this. Patients reporting pain and discomfort six months post-injury had an almost ten-fold higher risk of future arthroplasty compared to those with no pain. We acknowledge that this may be an intuitive finding for those who commonly treat patients undergoing revision arthroplasty. However, this is the first study to provide empirical evidence of the association between pain and revision arthroplasty after hip fracture and the first to quantify the magnitude and timing of that association. Furthermore, this study has also demonstrated which PROMs may have less predictive capacity for future revision, specifically mobility, self-care, usual activities and anxiety/depression. Consequently, our findings reinforce the importance of pain as a critical outcome to collect, and act on, following femoral neck fracture.

## Figures and Tables

**Table 1:** Characteristics of included patients (n = 2325) and factors associated with future revision arthroplasty/conversion to arthroplasty.

Characteristic	Patients with No Future Arthroplasty n (%)	Patients Undergoing Future Arthroplasty n (%)	*p*
Age group (years)			<0.001
55–64	134 (89.9)	15 (10.1)	
65–74	331 (94.0)	21 (6.0)	
75–84	857 (96.5)	31 (3.5)	
85+	926 (98.9)	10 (1.1)	
Gender			0.30
Male	618 (97.3)	17 (2.7)	
Female	1630 (96.5)	60 (3.5)	
Pre-injury disability ^1^			<0.001
None	769 (94.2)	47 (5.8)	
Disability reported	1257 (98.0)	26 (2.0)	
Charlson Comorbidity Index at time of injury			<0.001
0	1165 (95.4)	56 (4.6)	
≥1	1083 (98.1)	13 (1.9)	
Type index surgery			<0.001
Cemented hemiarthroplasty	1015 (98.5)	16 (1.5)	
Cementless hemiarthroplasty	345 (97.2)	10 (2.8)	
Cemented total arthroplasty	79 (95.2)	4 (4.8)	
Cementless total arthroplasty	67 (95.7)	3 (4.3)	
Internal fixation	742 (94.4)	44 (5.6)	
Total	2248 (96.7)	77 (3.3)	-

^1^ Data missing n = 226 (9.7%).

**Table 2:** Six-month patient-reported outcome measures associated with future revision arthroplasty or conversion to arthroplasty (n = 2325).

Patient-Reported Outcomes	Patients with No Future Arthroplasty n (%)	Patients Undergoing Future Arthroplasty n (%)	*p*
Residence at 6 months post injury ^1^			<0.001
At home without additional care	357 (94.0)	23 (6.1)	
At home with additional care	800 (95.1)	41 (4.9)	
Nursing home/other	890 (99.2)	4 (0.8)	
Problems with mobility (EQ-5D) at 6 months post injury ^2^			0.28
No	257 (97.7)	6 (2.3)	
Yes	1592 (96.4)	59 (3.6)	
Problems with personal care (EQ-5D) at 6 months post injury ^3^			<0.01
No	628 (95.2)	32 (4.9)	
Yes	1177 (97.7)	28 (2.3)	
Problems with usual activities (EQ-5D) at 6 months post injury ^4^			0.89
No	374 (96.6)	13 (3.4)	
Yes	1460 (96.5)	53 (3.5)	
Pain and discomfort (EQ-5D) at 6 months post injury ^5^			<0.001
No	842 (99.4)	5 (0.6)	
Yes	989 (94.0)	63 (6.0)	
Anxiety (EQ-5D) at 6 months post injury ^6^			0.35
No	1043 (96.9)	33 (3.1)	
Yes	747 (96.1)	30 (3.9)	
Total	2248 (96.7)	77 (3.3)	-

^1^ Data missing n = 207 (8.9%); ^2^ Data missing n = 411 (17.7%); ^3^ Data missing n = 460 (19.8%); ^4^ Data missing n = 425 (18.3%); ^5^ Data missing n = 426 (18.3%); ^6^ Data missing n = 472 (20.3%); EQ-5D, European Quality of Life-Five Dimensions measure.

**Table 3:** Factors associated with future revision arthroplasty/conversion to arthroplasty, adjusted for confounding (only participants with no covariates missing, n = 1836).

Covariate	HR	95% CI	*p*
Age group (years)			<0.01
55–64	1.00 (ref)		
65–74	0.85	0.42, 1.73	
75–84	0.49	0.25, 0.95	
85+	0.22	0.09, 0.55	
Pre-injury disability			<0.01
None	1.00 (ref)		
Disability reported	0.47	0.29, 0.78	
Index surgery			0.07
Cemented hemiarthroplasty	1.00 (ref)		
Cementless hemiarthroplasty	1.67	0.72, 3.84	
Cemented total hip arthroplasty	1.64	0.53, 5.01	
Cementless total hip arthroplasty	1.24	0.35, 4.35	
Internal fixation	2.41	1.31, 4.45	
Pain and discomfort (EQ-5D) at 6 months post-injury			<0.001
No	1.00 (ref)		
Yes	9.50	3.81, 23.67	

EQ-5D: European Quality of Life-Five Dimensions measure; HR: hazard ratio.

## Supplementary Materials

The following are available online at https://www.mdpi.com/1660-4601/16/8/1325/s1, Table S1. Profile of patients with incomplete six-month follow up data; Table S2: (A) Univariable associations with future revision arthroplasty or conversion to arthroplasty (Included participants with date of injury 2009 onwards, n = 2028); Table S2: (B) Factors associated with future revision arthroplasty or conversion to arthroplasty, adjusted for confounding (Included participants with date of injury 2009 onwards, no covariates missing (n = 1762).
